# Mapping the Spatial Distribution and Characteristics of Lineaments Using Fractal and Multifractal Models: A Case Study from Northeastern Yunnan Province, China

**DOI:** 10.1038/s41598-017-11027-0

**Published:** 2017-09-05

**Authors:** Chunzhong Ni, Shitao Zhang, Zhong Chen, Yongfeng Yan, Yujian Li

**Affiliations:** Faculty of Land Resource Engineering, Kunming University of Science and Technology, Kunming, 650093 China

## Abstract

This study describes the fractal dimensions of the spatial distributions of lineaments as an index of the complexity of faults, and the results could provide new insights into the migration of ore-bearing fluid. The Segment Tracing Algorithm method is employed to extract the lineaments in northeast Yunnan Province from a remote sensing image. Box-counting fractal and multifractal models are used to analyze the fractal and multifractal spatial distribution characteristics of the linear structures. The different directions of the linear structure fractal dimensions are similar in the study area. The fractal dimensions of all lineaments, northeast trending lineaments and northwest trending lineaments are 1.98, 1.94 and 1.95, respectively. The dimensions of four large ore deposit fields, Kuangshanchang, Qilinchang, Maozu, and Lemachang, are 1.93, 1.92, 1.95, and 1.93, respectively. The fractal dimensions of these four fields are greater than those of the South China lineaments. The super-large and large ore deposits are consistent with fractal dimensions with high values. The scale index and the singular index show nonlinear relationships with any real number, and the fractal dimension spectrum has a unimodal curve. This study provides a useful reference for deposit exploration in areas with topographies similar to that in northeastern Yunnan.

## Introduction

A variety of methods are used to explore metal deposits, including geological mapping, geophysics, geochemistry, remote sensing geology, and tectonogeochemistry^1–7^. Tectonogeochemistry is a discipline used to describe the relationships among tectonic movement, evolution, and geochemical phenomena. Studies of faults (structures) represent an important focus of geochemistry studies, and these structures have been the subject of considerable research^8–11^.

Extensive faults are observed in the crust and develop when a rock or rock mass has experienced a significant displacement along the rupture surface. In the landform, large faults often form rifts and cliffs. Faults represent the connecting channel between the deep ore-forming substance and the ore body, and the spatial position of a fault can guide predictions of the ore body location. Researchers have studied the characteristics of faults using different methods. Fractal geometry is useful for studying fault systems, and the fractal dimensions of faults are widely used. Faults with high fractal dimensions are usually associated with mineral or hydrocarbon deposits^12^. Fractal geometry is used to analyze the relationship between the spatial distribution of fault systems and the distribution of oil in the southern part of the South China Sea^13^. The fractal dimensions of faults are also used to estimate the location of deposits and concluded that the known mineral deposits are located in high-value fractal dimension areas^14^. However, using faults for mineral exploration requires field measurements, which present high workforce and resource costs. Moreover, reliable data for specific types of terrain are difficult to obtain, and field measurements are often subjective. Therefore, an alternative method for mineral exploration is required.

Lineaments are straight lines or approximately linear landforms that are distributed along the surface of the earth, and their presence can be inferred indirectly from their influence on topography and magmatic activity. Lineaments are closely related to unexposed faults^15^. Most large faults are directly interpreted from lineaments observed in remote sensing images, and this method is efficient and limits anthropogenic interference. At present, many scholars have used these advantages associated with lineaments to elaborate on the relationship between lineaments and mineral exploration. Many hydrothermal ore deposits display a spatial relationship with large-scale lineaments and crustal discontinuities^16, 17^. The roles of two major crustal lineaments can be determined based on the formation of different types of ore deposits^18^. The regional lineaments of Iran have been identified using geographic information system (GIS) software to explore metallic ore deposits^16^. Many hydrothermal ore deposits have been found to display a spatial relationship with large-scale lineaments and crustal discontinuities^17^. The occurrence of banded iron formations in the Ogbomoso area of southwestern Nigeria was investigated using lineaments obtained from high-resolution aerial photos^19^. The potential trends of ores in Finland were analyzed using lineament data^20^. The prominent orientations of 206 fracture lineaments in the Ikom-Mamfe Basin of Nigeria were analyzed, and sphalerite, pyrite, and amethyst were found in the vein lineament^21^. Regions with high fractal dimensions represent zones with high probabilities of having well-connected faults and fluid pathways^22^. However, the current research on the relationship between lineaments and deposits is spatially qualitative. Current methods are unable to provide accurate quantitative forecasts of the spatial locations of mineral deposits and the size of ore bodies according to the spatial distribution characteristics of the lineament. Therefore, additional systematic research is required.

The northeastern region of Yunnan Province consists of two prefecture-level cities, Qujing and Zhaotong, and lies approximately 250 km away from Kunming, the capital city. This region is characterized by rough terrain and presents an altitude difference of 2500 m between the highest and the lowest points. Because of the complex topography of this region, the spatial distribution of Pb and Zn deposits is complex. Using the northeastern region of Yunnan Province as an example, this study applied the fractal dimensions and spatial distributions of lineaments as an index of the complexity of faults to identify an efficient and straightforward approach for predicting the migration of ore-bearing fluid. Therefore, this study used fractal and multifractal models and calculated the lineament fractal dimensions. A dimension contour map and trend surface contour map of the lineaments were drawn. Afterward, the lineament fractal dimension values and the discovered mineral deposits in the investigated region were compared.

## Results and Discussion

### Fractal dimensions of lineament patterns

The mapped lineaments in the study area were extracted using the segment tracing algorithm (STA) method and Landsat-7 ETM satellite images of the study area^23, 24^.

**Figure 1:**
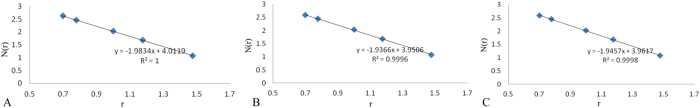
Log-log plots of cell size r versus the number of cells N(r) occupied by (**A**) all faults, (**B**) NE-trending faults, and (**C**) NW-trending faults.

The number of cells N(r) occupied by lineaments was counted in the raster map (Table 1). The values of D for all lineaments, NW-trending lineaments, and NE-trending lineaments were 1.98, 1.95, and 1.94, respectively, and the corresponding coefficients were 1, 0.999, and 0.999, respectively (Fig. 1). The results between r and N(r) exhibit a high correlation. The spatial distributions of lineaments in the study area are statistically similar. The zones with high indices of fractal dimensions are mainly located near Kuangshanchang, Qilingchang, Maozu and Lemachang. The fractal dimensions of the spatial distributions of lineaments in the four Pb-Zn deposits are 1.93 (Kuangshanchang), 1.92 (Qilingchang), 1.95 (Maozu) and 1.93 (Lemachang) (Table 2). The four known ore deposits in the study area are located in zones with high fractal dimensions. The value of D for the map of all lineaments and the value of D for the four ore deposits are greater than the fractal dimension values of the lineaments in the Shuiyanba W-Sn ore field^14^. The fractal dimension values of the lineaments in the Dexing porphyry copper ore field and the Gaolong gold deposit are 1.60 and 1.66, respectively (Table 1). The fractal dimensions of the study area indicate that the faults have a more complex structure and stronger activity than those of the other three areas.

**Table 1:** Results of the fractal statistics.

All		NE-trending		NW-trending	
Scale (km)	Grid number	Scale (km)	Grid number	Scale (km)	Grid number
5	420	5	385	5	392
6	293	6	277	6	280
10	108	10	107	10	106
15	48	15	48	15	48
30	12	30	12	30	12

**Table 2:** Fractal characteristics of 1225 lineaments in the study area and other areas.

Area	Fractal dimension	Data source
All lineaments	1.98	This study
The NW lineaments	1.95	This study
The NE lineaments	1.94	This study
Kuangshanchang Pb-Zn field	1.93	This study
Qilingchang Pb-Zn field	1.92	This study
Maozu Pb-Zn field	1.95	This study
Lemachang Pb-Zn field	1.93	This study
Shuiyanba W-Sn ore field	1.35	Liao *et al*. (2012)
Dexing porphyry copper ore field	1.60	Jin *et al*. (1998)
Gaolong gold deposit	1.66	Yang *et al*. (2005)

**Figure 2:**
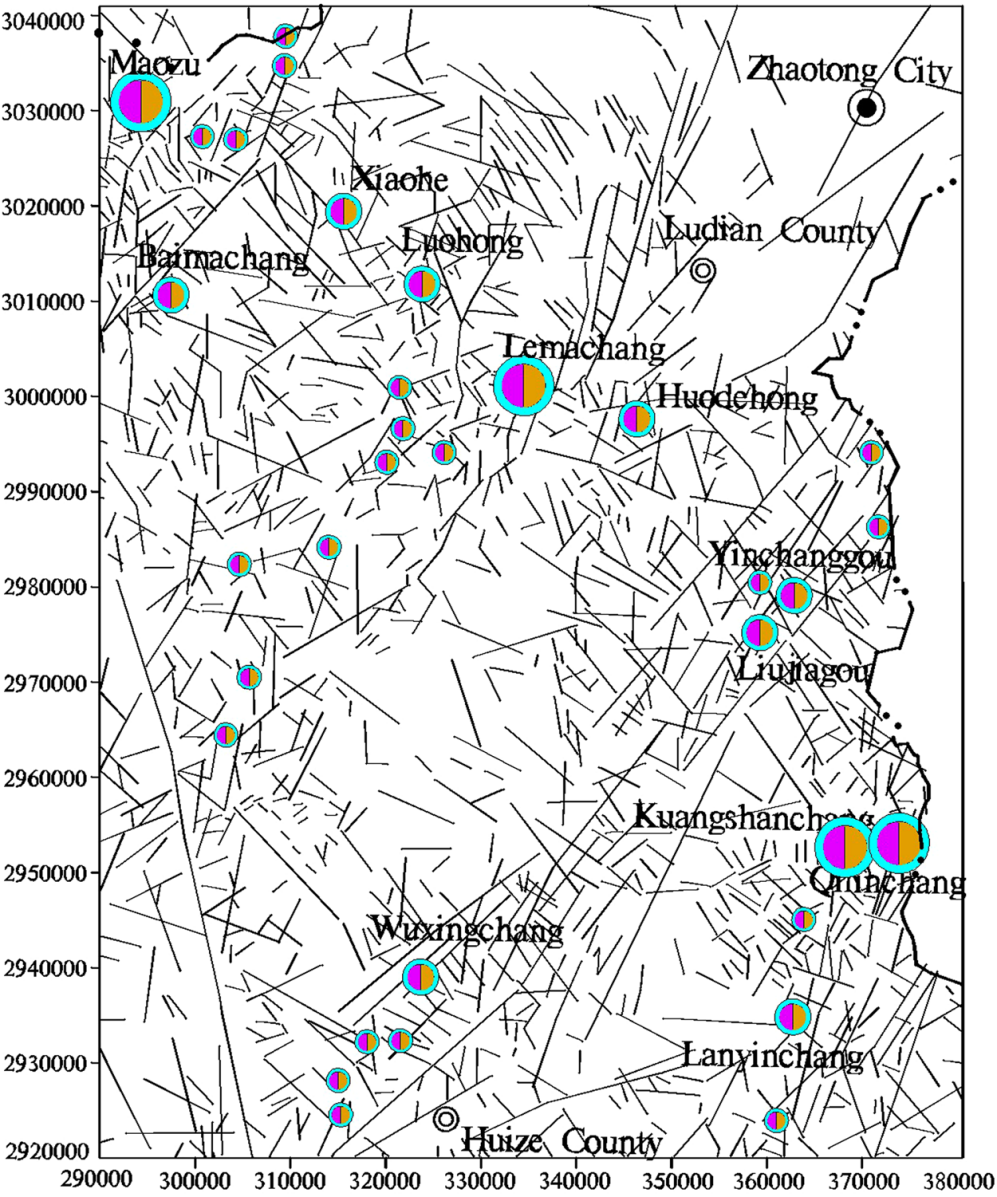
Lineaments extracted from remote sensing images of the study area (plotted with MapGIS 6.5, based on the Landsat-7 ETM satellite image of the study area, http://www.gscloud.cn/sources/list_dataset/241?cdataid=263&pdataid=10&datatype=L7slc-off#dlv=Wzg4LFswLDEwLDEsMF0sW1siZGF0YWRhdGUiLDBdXSxbXSw5OV0%3D).

**Figure 3:**
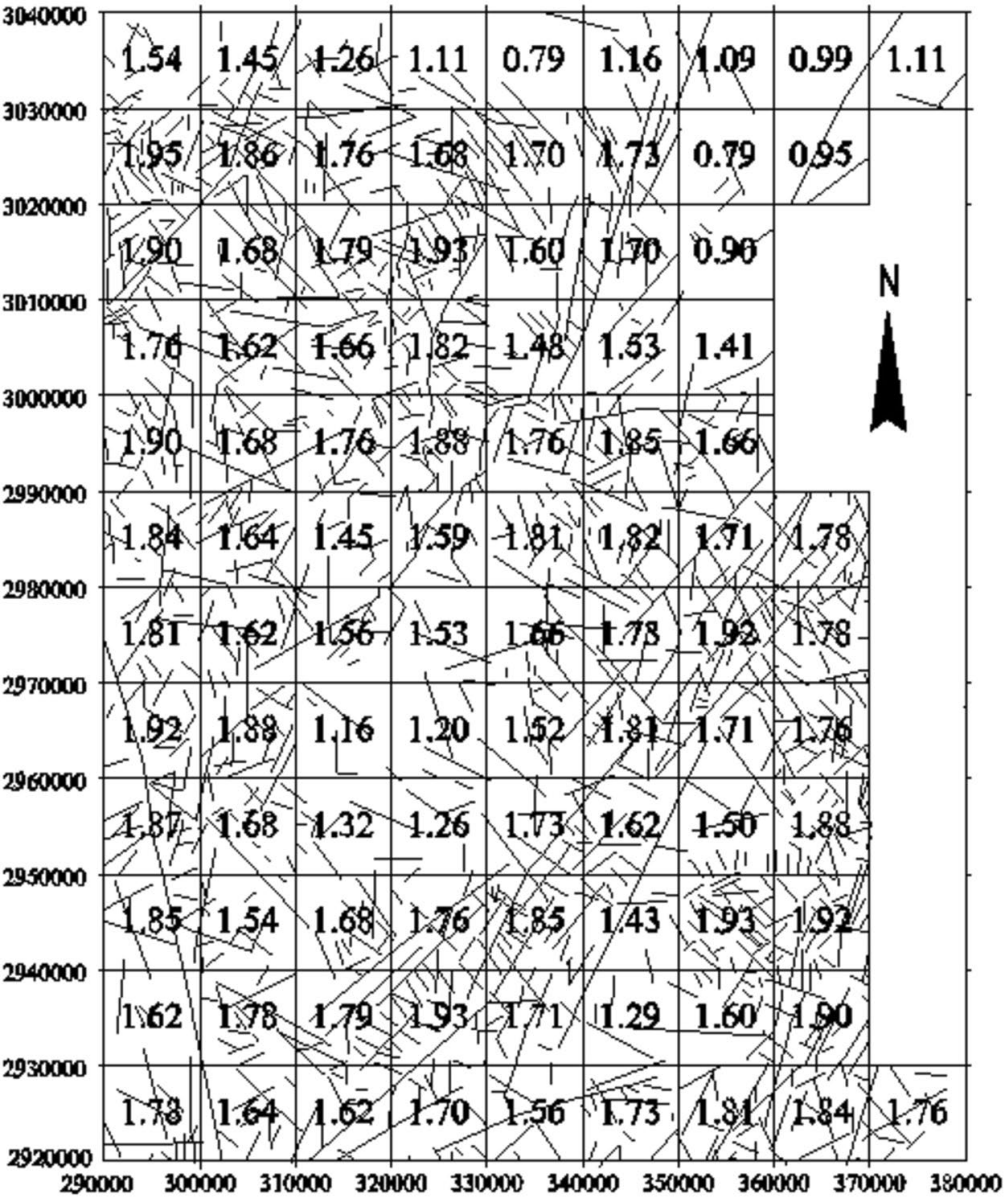
Estimates of the fractal dimensions of the spatial distributions of faults in each 10 × 10 km^2^ cell in the study area (plotted with MapGIS 6.5, based on the Landsat-7 ETM satellite image of the study area and the current experiment).

**Figure 4:**
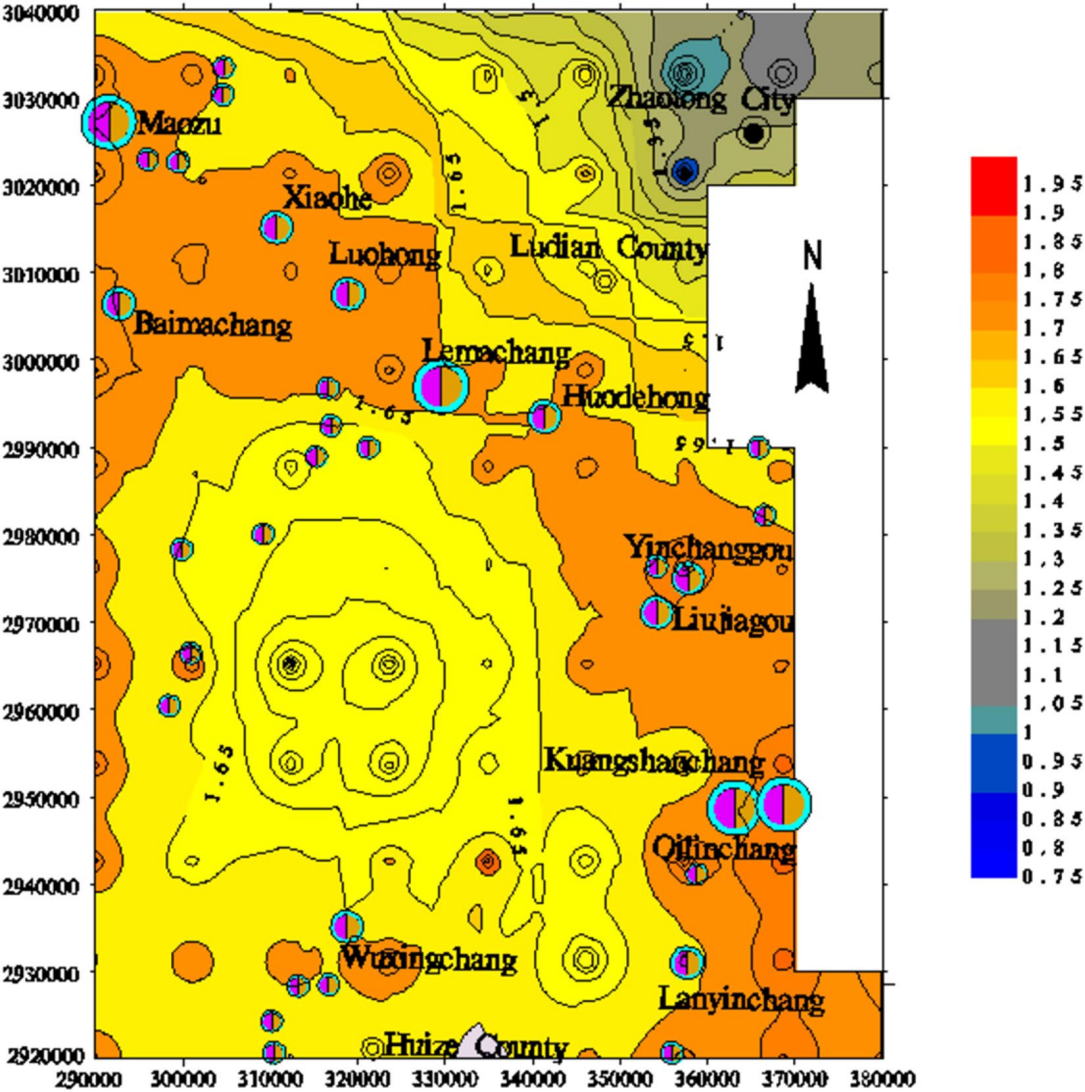
Contour map of the fractal dimension of the lineaments in the study area (plotted with MapGIS 6.5, based on the Landsat-7 ETM satellite image of the study area and the current experiment).

To further characterize the dimensional distribution of the lineaments in northeast Yunnan, the fractal dimensions of the lineaments in the digital image were calculated in 10 × 10 km^2^ grid cells in the study area using the box-counting fractal model. The 10 × 10 km^2^ grid cell size was selected for the extracted lineaments map according to the image scale (Fig. 2). The box sizes used for measuring the fractal dimensions in each 10 × 1 km^2^ cell were 10, 5, 2.5, and 1.25 km. The analysis of the fractal dimension of each cell suggests that higher numbers and densities of lineaments correspond to higher fractal dimensions (Fig. 3). The calculated fractal dimensions of cells containing faults were then interpolated to obtain a contour map of the fractal dimensions (Fig. 4).

The contour map of the fractal dimensions of the study area shows that the fractal dimension of lineaments is closely related to the number of lineaments. Lineaments with high D values (D > 1.70) mainly occur in the NW-trending belt, with an average width of 10 km from Qilinchang to Maozu. The low-value area (D < 1.00) is primarily distributed in the northeast region of the study area, where deposits seldom occur. In addition, the area with high fractal dimensions is more conducive to mineralization based on the contour map.

### Lineament fractal dimension trends

**Figure 5:**
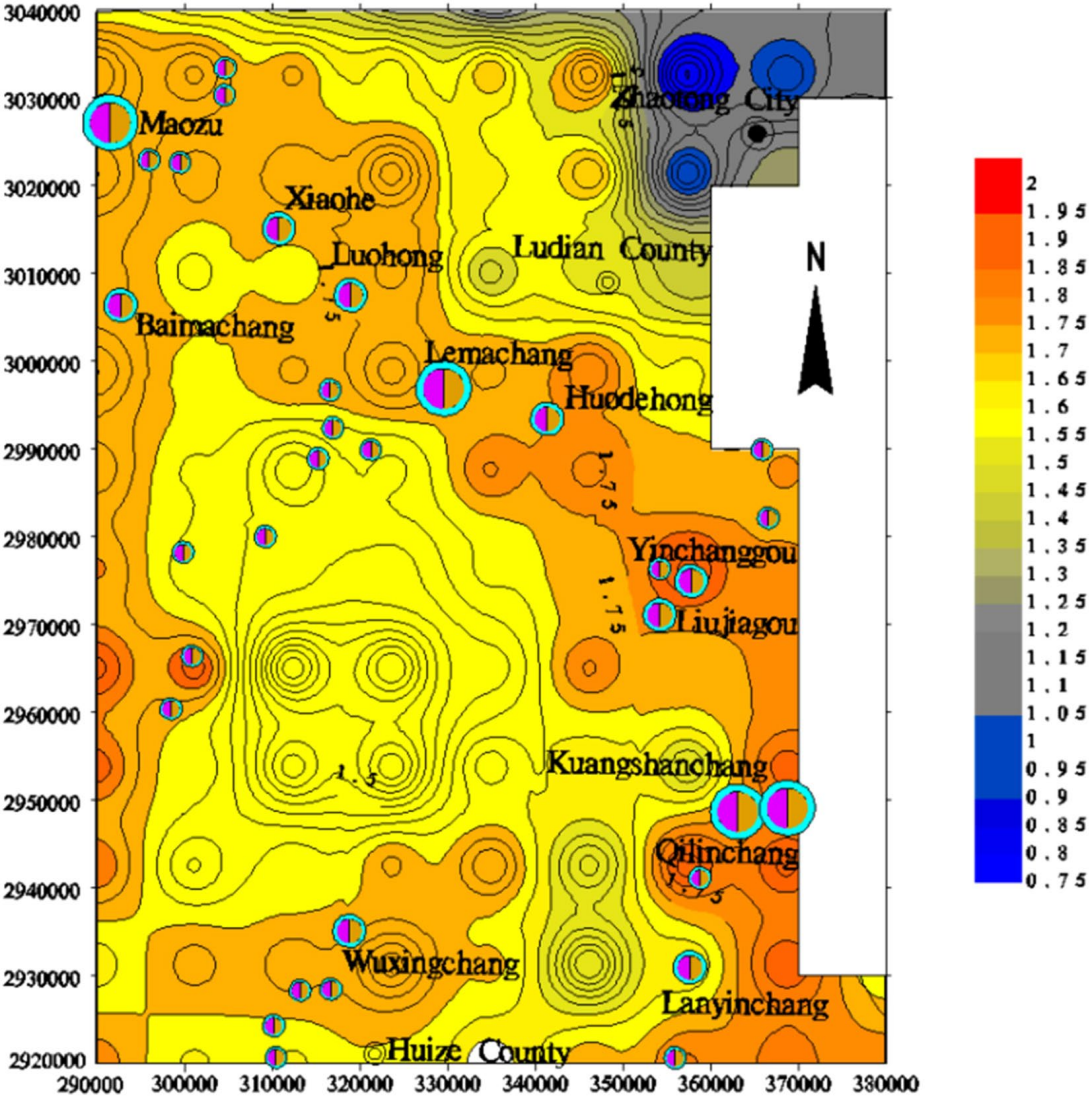
Two-step tendency map of the fractal dimensions of the lineaments in the study area (plotted with MapGIS 6.5, based on the Landsat-7 ETM satellite image of the study area and the current experiment).

The trend analysis shows that the zones with high fractal dimensions are well correlated with the deep faults in the contour maps of the fractal dimensions, indicating the potential existence of a deep fault that controls the development of the rock mass and ore-forming fluid migration (Fig. 5). The super-large and large deposits, such as the Qilinchang, Liujiagou, Yinchanggou, Lemachang, Luohong, Xiaohe, and Maozu deposits, are mainly located in the high fractal dimension area, which is near the NW-trending portion of the study area.

**Figure 6:**
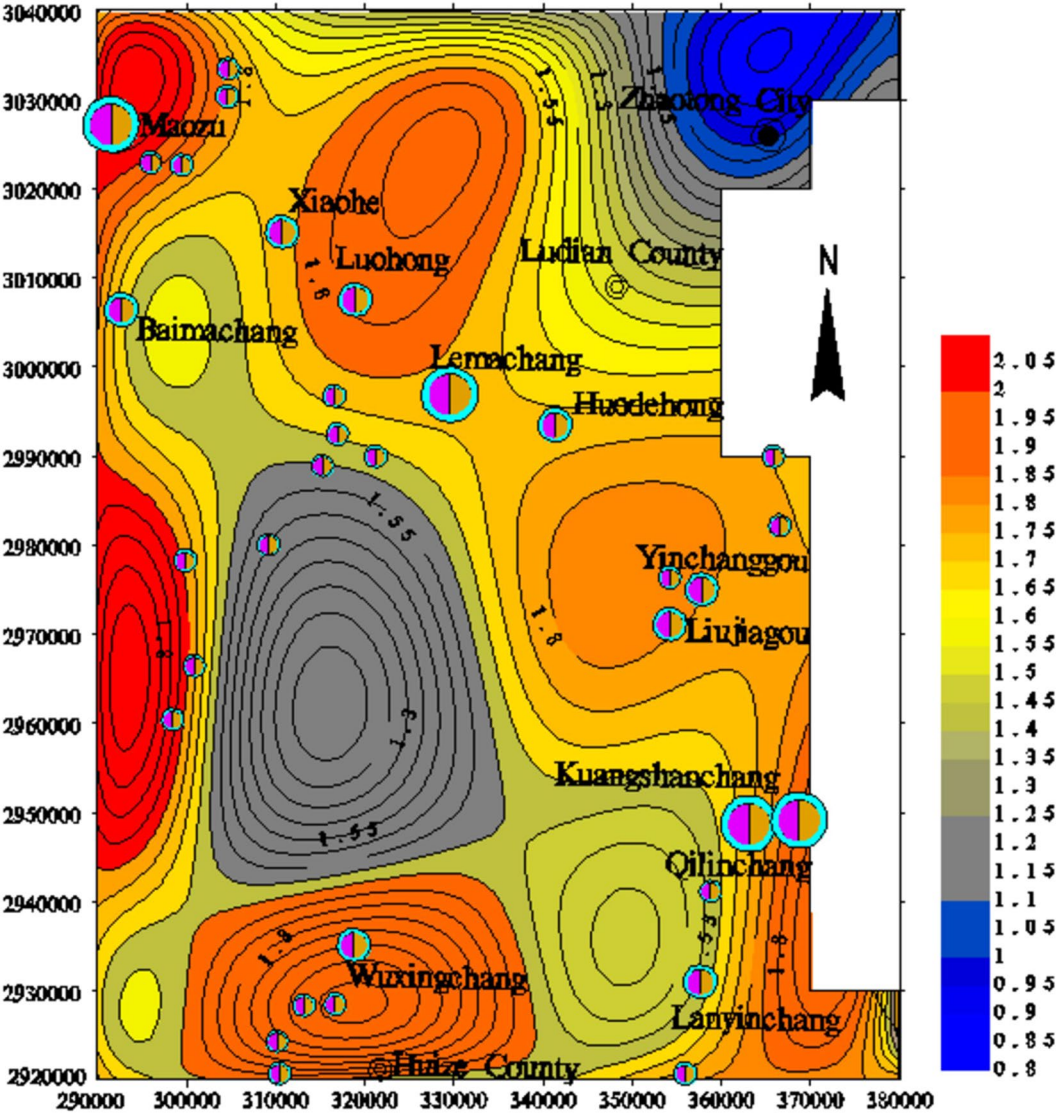
Six-step tendency map of the fractal dimensions of the lineaments in the study area (plotted with MapGIS 6.5, based on the Landsat-7 ETM satellite image of the study area and the current experiment).

The high-step tendency maps of the fractal dimensions of the lineaments show that the zones with high indices exhibit NW and NS orientations, especially in the 6-step tendency map (Fig. 6). Few lineaments with nearly NE orientations are observed, which indicates that NW-trending lineaments are more complex and vigorous than those with other orientations.

### Multifractal characteristics

To further describe the characteristics of lineaments in the investigated area, multifractal models are applied to the complexity of lineaments.

**Figure 7:**
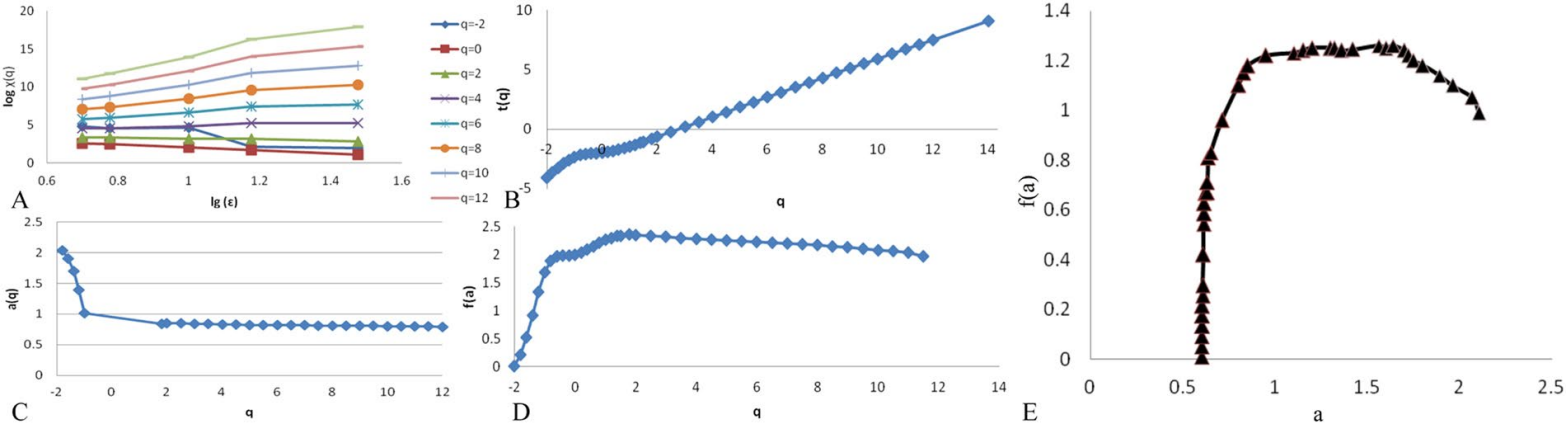
Results of the multifractal analysis: (**A**) log-log plots of ${\chi }_{q}(\varepsilon )$ versus $\varepsilon$; (**B**) estimates of $\tau (q)$, including the slopes of the straight lines in A; (**C**) singularity $\alpha (q)$ estimated from (**B**) using the central differentiation method; (**D**) multifractal spectrum $f(\alpha )$ estimated from $\tau (q)$, $\alpha (q)$, and q; and (**E**) plots of the multifractal spectrum $f(\alpha )$ versus $\alpha$.

For the multifractal modeling of the spatial distribution of faults, a log–log plot of ${x}_{q}(\varepsilon )$ and $\varepsilon$ is obtained with straight lines for small $\varepsilon$ (Fig. 7A). Values of $\tau (q)$ were estimated from those straight lines using least squares fitting with a standard error of less than 0.05 and an R^2^ value of greater than 0.99 (Fig. 7B). The singularity exponent $\alpha (q)$ was then derived by the central numerical differentiation of $\tau (q)$ (Fig. 7C and D). Finally, the multifractal spectrum $f(\alpha )$ was calculated from a Legendre transform (Fig. 7E).

In Fig. 7C and D, $f(\alpha )-q$ and $\alpha (q)-q$ do not exhibit simple linear relationships. The fractal dimension spectrum displays a curved distribution in Fig. 6E, which shows that the distribution of lineaments in the study area is multifractal and can be used to guide fluid migration and ore body prediction.

## Conclusions

The lineaments in the northeastern region of Yunnan Province were extracted from images using the STA method, and both the fractal and multifractal features were used to map the spatial distribution and characteristics of the lineaments in the study area. Many scholars have conducted fractal geometry research on faults, although few researchers have considered lineaments. Because of the close relationship between lineaments and faults, the spatial distributions of lineaments and mineral deposits were analyzed using fractal theory in this paper. This method is beneficial for preinvestigations of minerals in dangerous and execrable areas, and it provides a cost-effective and rapid method for mineral exploration.

The results show that the fractal dimensions of lineaments are directly related to the degree of development of fault structures and the spatial distribution of ore bodies. The lineaments in the four large deposits are fractal, and the fractal structure exhibits statistical self-similarity. The fractal dimensions of the four super-large ore deposits were high and suggest that a high fractal dimension corresponds to the occurrence of mineralization. The lineament fractal dimension contour map and trend surface contour map show that the linear structures of the lineaments in the area are NW-trending and NS-trending; moreover, the ore bodies that have been identified in this region occur along these fractal dimensions in high-value areas. The multifractal charts illustrate that the local lineaments obey a multifractal distribution, which may lead to the migration of ore fluid with multifractal characteristics; therefore, the results can be used as an index for ore body prediction.

The method presented in this study is efficient and allows for the automation of lineament extraction, which can reduce the associated error margins.

## Methods

### Geological setting

The Pb-Zn metallogenic region in northeastern Yunnan Province (center coordinates: 27 degrees north latitude and 103 degrees east longitude) is located in a passive continental margin of the Yangtze subplate. Multiphase tectonic and intrusive activities have generated unique Ge, Pb, and Zn concentrations in the area and formed the large Ge, Ag, Pb, Zn deposits.

Three faults are located within the metallogenic area of the province: the NS-trending crust-cutting deep fault of Xiaojiang, the NE-trending Shizong-Mile fault, and the NW-trending Kangding-Yiliang-Shuicheng fault. The Kangding-Yiliang-Shuicheng fault is located in the northwest part of the area and consists of a series of northwest-oriented fractures and fold structures. These northwest tectonic belts that stretch hundreds of kilometers are major ore-control structures and display wide distributions of Pb-Zn deposits and mineral points. The nearly NS orientation of the Maoping-Qujing-Zhaotong conceals fault located in the center of the area and is also an important ore-controlling structure. There are also many NW orientated secondary faults and folds stretching for several kilometers, such as the Kuangshanchang-Luohong-Maozu fault and the Yinchang-Wuxingchang fault (fold), which are composed of a series of northwest fractures and folds. Many Pb-Zn deposits (points) are distributed along these tectonic belts.

**Figure 8:**
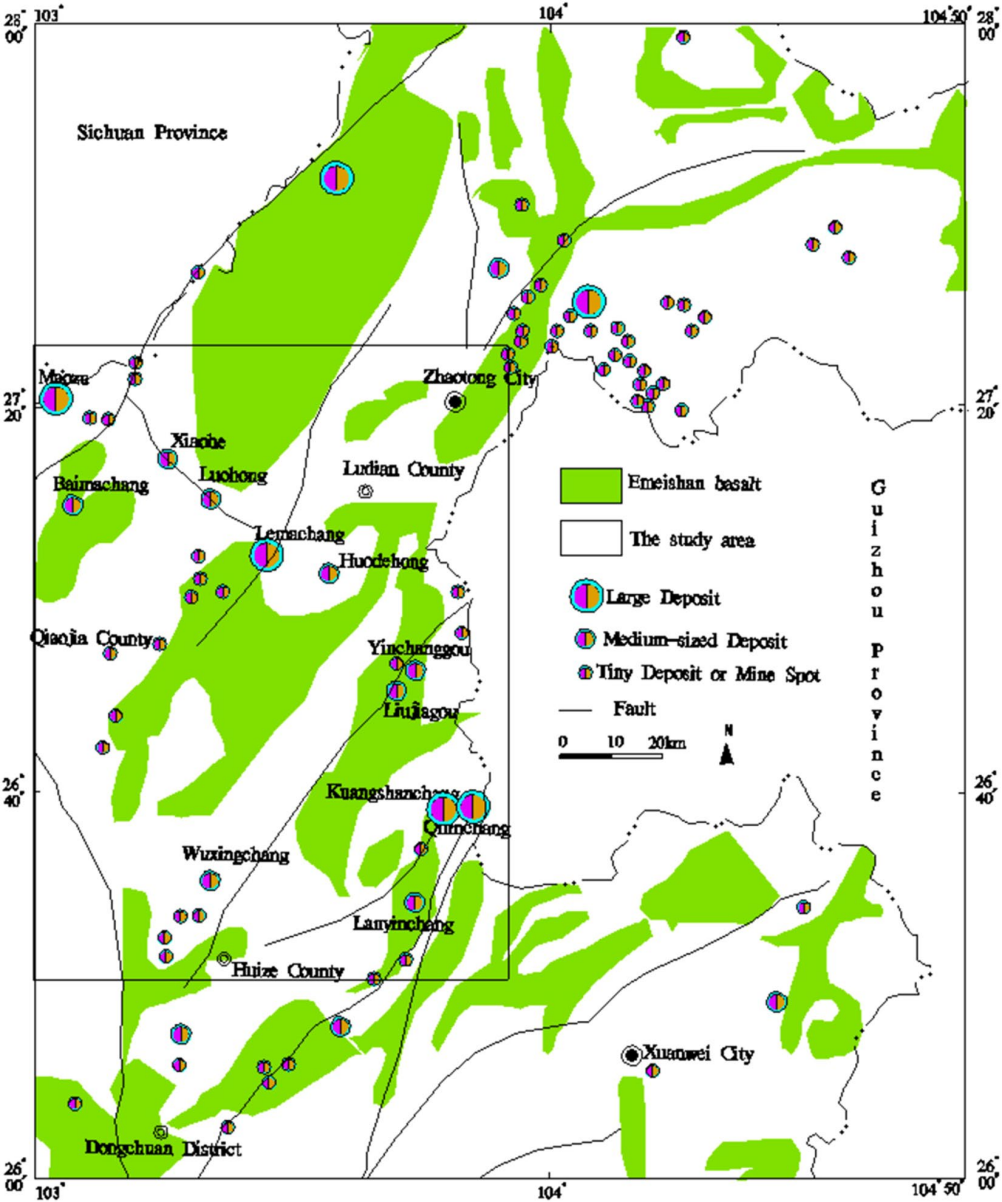
Distribution of the mineral deposits, Emeishan basalt and the main lineament in the study area (plotted with MapGIS 6.5 and Excel, based on the Landsat-7 ETM satellite image of the study area and the current experiment).

The extrusive rock in the study area contains mid-subsiliceous rocks of the Mesoproterozoic Kunyang Group, Sinian volcanic tuff and Permian Emeishan basalt. Emeishan basalt is the main magmatic rock type (Fig. 8)^25^, and the Emeishan basalt extension is consistent with a series of striking thrusts.

### Background theories

#### Box-counting fractal model

Many methods are used to calculate fracture fractal dimensions, such as circle covering, length-frequency statistics, sliding windows and box dimensions. One of the most commonly used methods is the box dimension method^26–29^.

When all of the extracted lineaments in the study area are identified in a raster map, statistical analyses of the fracture information are conducted using the initial cells with a side length L. Using a small square grid with a side length r equal to L/2^n^ (where n is an integer), the fracture trace number $N(r)$ contained in the corresponding scale grid is obtained. When r is small enough, the required minimum number of cells between $N(r)$ and r satisfy the following formula:

1 $$N(r)\propto k{r}^{-D}$$

where D is a box-counting fractal dimension from 0 to 2 in the two-dimensional map, k is a constant, r is a measure of unit size, $N(r)$ is the cumulative number of cells containing lineaments, D is a box-counting fractal dimension and $\propto$ represents a proportionality. By plotting $\mathrm{lg}\,r-\,\mathrm{lg}\,N(r)$ on a graph in the coordinate system, a straight-line regression is obtained by least-squares fitting and the absolute value of the regression coefficient from the fractal dimension values of D. The determination coefficient R^2^ can be obtained simultaneously. R^2^ values closer to 1 indicate a better degree of fit and a greater conformance of the fractal graphics to the scale relationship in equation (1).

#### Multifractal models

To further describe the characteristics of lineaments in the study area, multifractal models are applied to the complexity of lineaments. A box-counting fractal model is constructed by creating a square grid with a defined side length in the lineament raster map. When a lineament is located in the corresponding grid, the grid will be recorded. Thus, only grids that contain lineaments are involved in the calculation of the fractal dimension, and the fractal dimension is not influenced by the number of lineaments in the grid. Multifractal models can describe the number of lineaments in each scale grid, and the scale index and the fractal dimension index represent a continuous change. When the lineament number in each grid is calculated, a log–log plot of the lineament number and the corresponding grid side length is produced with straight lines. Then, the mass exponent can be deduced by least squares fitting. Finally, the multifractal characteristics of lineaments can be elucidated based on the line shape and the multifractal spectrum. Evertsz and Mandelbrot argued that fractal analyses should be applied to the collection of objects and multifractal analyses should be applied to the measurement of objects^30^. Fractal sets can be measured by determining their presence or absence in collections of cells created by the partitioning of k-dimensional space R^k^ (k = 1, 2, or 3)^31^. This method assumes that $\mu (L)$ represents the measurement R^k^ in the set L. In this article, L is the linear structure and $\mu {(L)}^{\ast }$ is the number of the linear structure. To study the multifractal properties of a linear structure, each fault map is converted into separate raster maps with different spatial resolutions $\varepsilon$. The number of lineaments in cell i with side lengths $\varepsilon$ is ${\mu }_{i}(\varepsilon )$. The partition function can be then defined as follows:

2 $$xq(\varepsilon )=\sum _{i=1}^{N(\varepsilon )}{\mu }_{i}^{q(\varepsilon )}$$

where $N(\varepsilon )$ is the total number of unit cells of size $\varepsilon$ and q is any real number. If $\mu (\varepsilon )$ satisfies the multifractal model, then the partition function ${x}_{q}(\varepsilon )$ and the size $\varepsilon$ satisfy a power-law relationship.

3 $${x}_{q}(\varepsilon )={\varepsilon }^{\tau (q)}$$

where $\tau (q)$ is the mass exponent of order q. If the lineaments meet the multifractal assumption, then a series of straight lines occur on the $\mathrm{log}\,{x}_{q}(\varepsilon )-log\varepsilon$ graph.

Multiple fractal dimension values $D(q)$ can be calculated using $\tau (q)$:

4 $${D}_{q}=\tau (q)/(q-1)$$

If r is smaller, then self-similarity can be described as follows:

5 $$u(r)={r}^{\alpha }$$

where α is a singular exponent. All of the cells with the same α compose a fractal set of f(α). In addition, $\alpha (q)$ is a function of q and α equals $\alpha (q)$ for multifractals with self-similarity. Formula (6) can then be obtained from (3) and (5)^30^.

6 $$\alpha (q)=\frac{\partial \tau (q)}{\partial q}$$

The value of $\alpha (q)$ is the same for all q in a single fractal, whereas for multiple fractals, the fractal dimension spectrum function $f(q)$ has the following form:

7 $$f(q)=q\alpha (q)-\tau (q)$$

where $\alpha (q)$ is a decreasing function of q and f(α) is a convex function of α. The ranges of these two functions and the curvature of function $\tau (q)$ indicate the degree of multifractality^27^.

### Experimental procedure

The mapped lineaments in the study area (Fig. 2) are extracted using the Segment Tracing Algorithm method (STA) from a Landsat-7 ETM satellite image of the study area^30, 31^. A total of 1,463 lineaments were extracted from an ETM+ image covering the Xiaohe-Kuangshanchang region (Fig. 2). This image was acquired on February 28, 2003 and presents less than 5.7% cloud cover, and the orbit number is p129/r44. The map of the studied lineaments covers approximately 110 km^2^, and the fractal dimension D in every cell is calculated using the box-counting method. The fractal analyses were conducted separately for the map that includes all of the lineaments and the maps of the NW and NE-trending lineaments. Each lineament map is converted into a separate raster map with spatial resolutions (r) of 5, 6, 10, 15, and 30 km. In each raster map, the number of cells N(r) occupied by lineaments is counted. Then, a log-log plot of N(r) versus r for each lineament map is constructed. Finally, a fractal dimension contour map and a trend surface contour map are plotted using the calculated fractal dimension of each cell.

### Verification

Fractal dimensions can reflect the degree of fracture of a rock mass, thereby directly indicating the migration of ore-bearing fluids according to the available research. Subsequent differentiation can cause ore-bearing fluids to form deposits of different sizes. Therefore, higher fractal dimension values correspond to areas that are more conducive to the formation of ore bodies and indicate a higher likelihood of identifying large deposits in the area, which increases the prospects for ore exploration. The transition zone from a high fractal dimension to a low fractal dimension is often the site where the stress changes rapidly, and small amounts of minerals usually accumulate and deposit in these locations. Therefore, medium-sized deposits and small deposits within transition zones should be the focus of ore bodies predictions, which is verified by our results because a relationship was observed between mineralization points and fractal dimensions.
